# Natural course of subclinical hyperthyroidism in primary care in the Netherlands

**DOI:** 10.1530/ETJ-25-0142

**Published:** 2025-11-18

**Authors:** Stan R Ursem, Wendy P J den Elzen, Jesse M van den Berg, Anita Boelen, Petra J M Elders, Raymond Noordam, Annemieke C Heijboer

**Affiliations:** ^1^ Endocrine Laboratory, Department of Laboratory Medicine, Amsterdam UMC, Location University of Amsterdam and Location Vrije Universiteit Amsterdam, Amsterdam, The Netherlands; ^2^ Amsterdam Gastroenterology, Endocrinology & Metabolism, Amsterdam, The Netherlands; ^3^ Department of General Practice, Location Vrije Universiteit Amsterdam, Amsterdam UMC, Amsterdam, The Netherlands; ^4^ Laboratory Specialized Diagnostics & Research, Department of Laboratory Medicine, Location University of Amsterdam, Amsterdam UMC, Amsterdam, The Netherlands; ^5^ Amsterdam Public Health Research Institute, Amsterdam, The Netherlands; ^6^ PHARMO Institute for Drug Outcomes Research, Utrecht, The Netherlands; ^7^ Department of Clinical Epidemiology, Leiden University Medical Center, Leiden, The Netherlands; ^8^ Health Campus The Hague/Department of Public Health and Primary Care, Leiden University Medical Center, The Hague, The Netherlands; ^9^ Amsterdam Reproduction & Development Research Institute, Amsterdam, The Netherlands

**Keywords:** subclinical hyperthyroidism, primary care, incidence, epidemiology, hyperthyroidism

## Abstract

**Background:**

Subclinical hyperthyroidism (SHT), a low serum thyroid-stimulating hormone (TSH) and normal free thyroxine (FT4) concentration, has potential health implications, yet the epidemiology and factors influencing its natural course in a primary care setting remain unclear.

**Objectives:**

To investigate the incidence and natural course of SHT in primary care and assess guideline adherence to follow-up recommendations within Dutch primary care.

**Methods:**

Using a retrospective cohort design in general practitioner data from the PHARMO Data Network in the Netherlands (2012–2021), patients with biochemically confirmed SHT were followed to assess progression to hyperthyroidism, recovery, or persistence. Adherence to the Dutch primary care SHT guideline was evaluated.

**Results:**

The SHT annual incidence was approximately 200 per 100,000 person-years. Among the 11,163 SHT patients, 47% recovered, 11% persisted, and 8% progressed to overt hyperthyroidism over a median follow-up of 5 years. Lower TSH (<0.1 mU/L) and female sex were associated with lower odds of recovery (OR for TSH <0.1 mU/L: 0.50, 95% CI: 0.43–0.58; OR for women: 0.82, 95% CI: 0.70–0.96) and higher odds of progression to overt hyperthyroidism (OR for TSH <0.1 mU/L: 2.36, 95% CI: 1.97–2.83; OR for women: 1.69, 95% CI: 1.32–2.17). Guideline adherence evaluation showed that 33% received follow-up TSH measurement within 6 months, and 4% underwent TSH-receptor antibody testing.

**Conclusion:**

This study highlights that a small subset of SHT patients progress to overt hyperthyroidism. Factors increasing the odds for progression included lower baseline TSH and female sex. Our findings indicate a need for improved guideline adherence.

## Introduction

Subclinical hyperthyroidism (SHT) is a condition characterized by a thyroid-stimulating hormone (TSH) concentration below the reference interval (RI) while the free thyroxine (FT4) concentration remains within the RI. This can result from endogenous causes, such as mild overproduction of thyroid hormones in Graves’ disease, toxic multinodular goiter, or thyroiditis, as well as from exogenous factors, such as excessive thyroid hormone supplementation (1). SHT is typically classified into two types: type 1, when TSH concentrations range between 0.1 and 0.4 mU/L, and type 2, when TSH concentrations fall below 0.1 mU/L (2). This has served as a critical threshold for both classification and treatment decisions (1).

SHT has been associated with an increased risk of atrial fibrillation, coronary artery disease, heart failure, osteoporosis, fractures, and mortality (3, 4, 5, 6). There is also evidence suggesting an increased risk of stroke, cognitive decline, and dementia, with evidence for stronger associations at lower TSH concentrations, although meta-analyses have shown inconsistent findings (7).

The prevalence of endogenous SHT varies considerably in the literature, ranging from 0.6 to 16%, influenced by diagnostic criteria, age, sex, type of TSH assay, and iodine intake (8). On average, a prevalence of 1–2% is reported, with higher prevalences observed in older people. Similar variation is observed in studies reporting the natural course of SHT. In earlier studies, the progression from subclinical to overt hyperthyroidism occurred in a relatively small proportion of individuals, with annual rates varying between 0.5 and 8%, depending on the underlying cause and population studied. Recovery rates in primary care settings have been reported to vary between 20 and 53%, with lower TSH levels associated with a lower recovery rate (9, 10). This variation may be explained by differences in cohort sizes, follow-up periods, and overrepresentation of certain subgroups, which limit the generalizability of findings (11). In addition, our recent systematic review of all available SHT guidelines highlighted inconsistencies and a general lack of evidence-based recommendations for follow-up (12).

Therefore, this study aimed to investigate the incidence and natural course of SHT in primary care in the Netherlands using a large longitudinal database of primary care patients with 10 years of follow-up and biochemically confirmed SHT in real-world data with confirmed generalizability (13). In addition, we explored factors associated with progression or recovery of SHT and assessed guideline adherence.

## Methods

### Study design and setting

We conducted a retrospective cohort study using data from the longitudinal Dutch general practitioner (GP) database from the PHARMO Data Network. Records include information on diagnoses and symptoms, laboratory test results, and prescriptions. Diagnoses were coded using the International Classification of Primary Care (ICPC), and prescriptions were coded using the WHO Anatomical Therapeutic Chemical (ATC) classification system. The GP data cover a catchment area representing 3.2 million residents (∼20% of the Dutch population) and include general practices from all regions of the Netherlands, with demonstrated representativeness in terms of age, sex, and urban–rural distribution (13).

The study was approved by the Institutional Review Board of Stichting Informatievoorziening voor Zorg en Onderzoek (STIZON, reference number CC2023-10, 30 March 2023).

### Time frame

Data from January 1, 2012, to December 31, 2021, were analyzed, encompassing a 10-year period to assess the incidence trends of SHT and its natural course.

### Incidence

A detailed definition of the population at risk and exclusion criteria is provided in the Supplemental information (see section on Supplementary materials given at the end of the article).

### Study population for dataset

**Figure 1:**
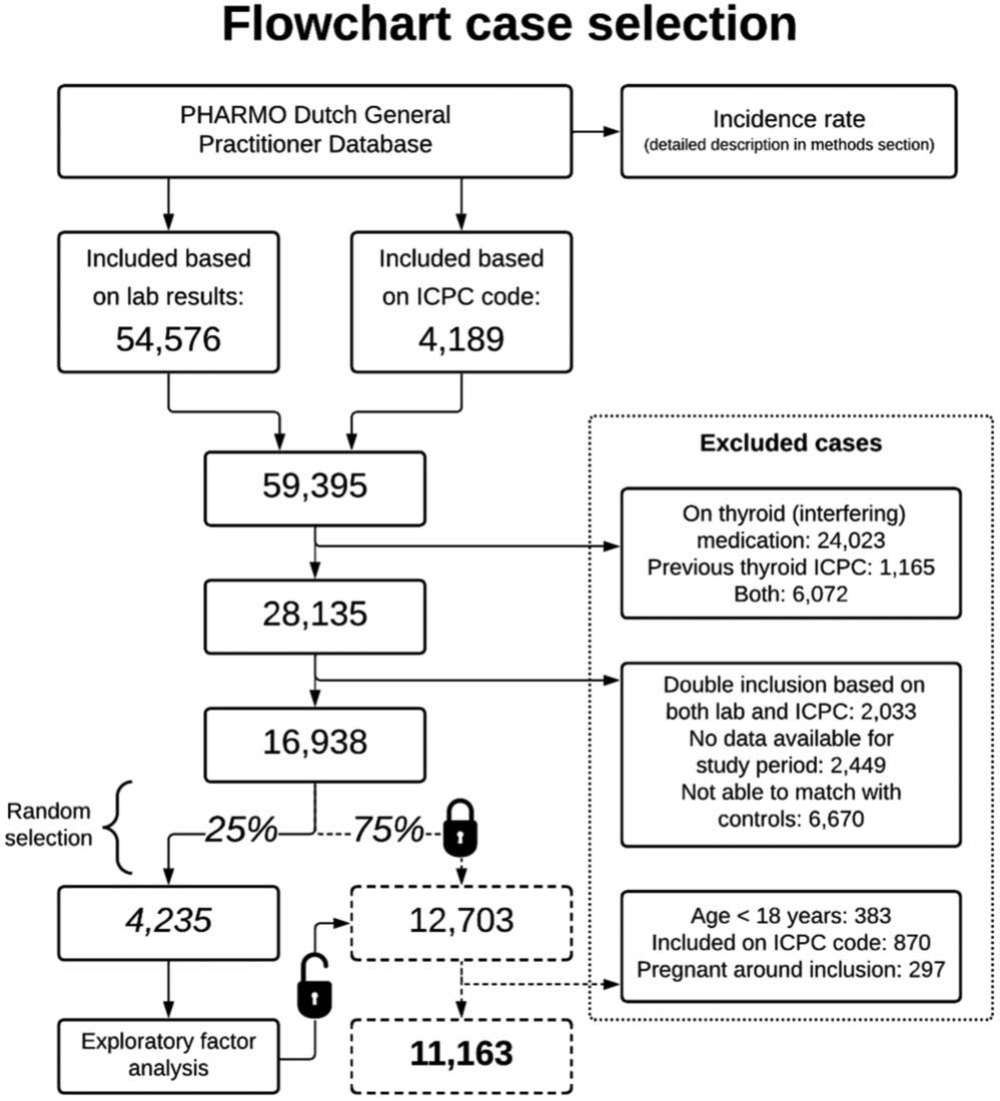
Flowchart of case selection.

The study population consisted of patients with SHT identified through primary care TSH measurements, excluding those with known thyroid diseases or conditions potentially interfering with thyroid function. Pregnant individuals and measurements around pregnancy (3 months before to 1 year after a registered pregnancy) were also excluded. A detailed overview of the patient selection process is presented in Fig. 1, which illustrates the flow diagram of the inclusion and exclusion criteria applied in this study. Additional detailed patient selection criteria are provided in the online supplements.

### Exploratory and confirmatory factor analysis

Exploratory factor analysis (EFA) was conducted on a randomly selected subset comprising 25% of the database with matched reference patients. The EFA helped to define the exact variables and develop robust outcome measures. After finalizing the analysis protocol based on the EFA results, the protocol was registered on the ISRCTN registry to maintain transparency and reproducibility (https://doi.org/10.1186/ISRCTN15496928) (14).

Once the analysis protocol was published, the remaining 75% of the database, which had been withheld to prevent bias, was decoded and made available for research. A confirmatory factor analysis (CFA) was subsequently performed on this larger dataset to validate the factors identified in the EFA and confirm the consistency and reliability of the defined outcome measures. The results shown in this article are from the 75% confirmatory factor analysis, except for the incidence data.

### Outcome measures

The following patient subgroups were identified, of which detailed definitions can be found in the supplement:

- Progression to overt hyperthyroidism: FT4 above RI.
- Progression to (subclinical) hypothyroidism: TSH above RI but FT4 within RI.
- Recovery: TSH normalized within RI.
- Persisting SHT: persistent TSH suppression with FT4 within RI.
- Unknown outcomes: missing follow-up TSH or FT4 measurements.

### Statistical analysis

To compare subgroups, we used ANOVA for continuous variables and chi-square tests for categorical variables. Logistic regression analyses were conducted to identify factors associated with progression and recovery. Independent variables included age, sex, TSH level at inclusion, and a comorbidity score based on cardiometabolic risk factors (e.g., hypertension, hypercholesterolemia, kidney disease, and diabetes). To reduce potential bias from incomplete follow-up, analyses of factors associated with recovery and progression were restricted to patients with known outcomes, using those with persisting SHT as the reference group. Comorbidity scoring methods and R packages and versions used for analysis are described in detail in the supplement.

### Guideline adherence

We assessed guideline adherence to the Dutch primary care guideline for thyroid disease by examining the frequency at which TSH and FT4 tests were performed during the follow-up period (excluding the first 4 weeks) (15). Furthermore, we evaluated adherence to recommended diagnostic workups, such as the measurement of TSH receptor antibodies (TRAb), erythrocyte sedimentation rate (ESR), or C-reactive protein (CRP), and leukocyte counts. In addition, we investigated the occurrence of non-recommended diagnostic tests, including TPO antibody measurements.

## Results

### Cohort characteristics

Figure 1 provides a flowchart of the case selection process. Initially, 59,395 cases were identified from PHARMO’s GP data based on laboratory results (54,576 cases) and SHT ICPC codes (4,189 cases). After exclusions for interfering factors (e.g. medication, previous thyroid conditions, and inability to match with controls), 16,938 cases were selected. The most frequent reason for exclusion was thyroid-interfering medication. The exclusion of individuals who could not be matched to controls was only relevant for a separate, linked study on clinical outcomes (https://doi.org/10.1186/ISRCTN15496928). For the EFA, 4,235 cases were included, in which we identified frequent misclassification of the SHT ICPC-included patients with a diagnosis of SHT where biochemistry showed subclinical hypothyroidism (14%). It was decided to exclude the ICPC-included patients in the confirmatory factor analysis and use only cases with a biochemical diagnosis of SHT. Ultimately, 11,163 SHT cases were included for the current analysis.

### Incidence

**Figure 2:**
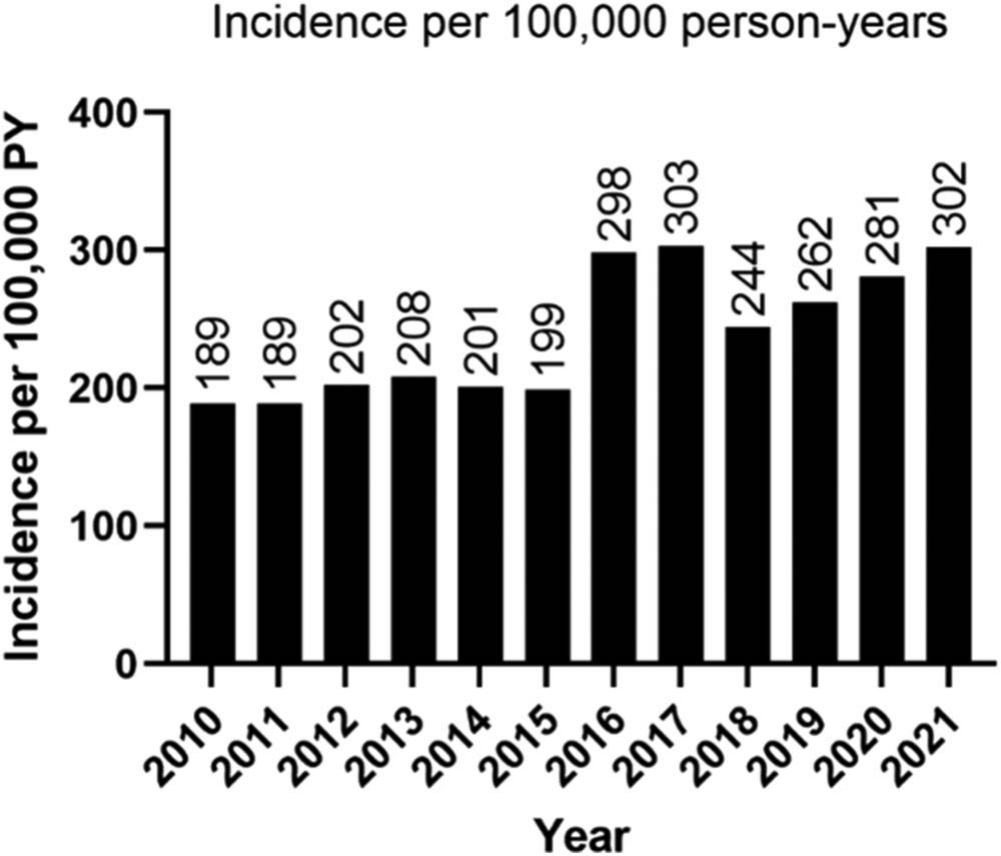
Incidence of subclinical hyperthyroidism per year in PHARMO GP data.

Figure 2 presents the annual incidence of SHT within the complete active PHARMO GP data. From 2010 to 2015, SHT incidence appeared stable at approximately 200 cases per 100,000 person-years, followed by a temporary and significant increase in 2016 and 2017, with an incidence of 300 per 100,000 person-years. From 2018 to 2021, the incidence incrementally and significantly increased over the years from 244 to 302 per 100,000 person-years. Additional data on trends and incidence rate ratios per year are available in Supplementary data S2. These changes did not coincide with changes in the RI or the total amount of TSH requests.

### Natural course outcomes

Table 1 provides a summary of the characteristics and outcomes observed across five subgroups. The median follow-up time of patients with SHT was 55 months. Nearly half of the patients achieved recovery (47%, 5,282), with their TSH levels returning to the RI during follow-up. After the recovery group, the group without known TSH follow-up data was the largest (29%), followed by the groups with persisting SHT (11%), progression to overt hyperthyroidism (8%), and transition to (subclinical) hypothyroidism (5%). The time until group definition had a wide interquartile range for all classifications. Notably, the median time until progression to hyperthyroidism (23 months, IQR: 8–49) was longer than the time until transition to (subclinical) hypothyroidism or recovery (respectively, 14 and 12 months, IQR: 3–41 and 5–28). The age of those who progressed to overt hyperthyroidism (62 years, IQR: 48–75) or who had persisting SHT (65 years, IQR: 52–75) tended to be higher than those who recovered (55 years, IQR: 40–69). Furthermore, a larger proportion of women were observed in all groups, and representation of women was particularly higher in the groups that progressed to hyperthyroidism or transitioned to (subclinical) hypothyroidism. The group with unknown outcomes included a slightly higher proportion of men and had higher baseline TSH values.

**Table 1:** Characteristics of subgroups of patients with subclinical hyperthyroidism. Data are presented as *n* (%) or as median (IQR).

Characteristics	Progression to HT	Transition to (S)HOT	Recovery	Persisting SHT	Unknown	Total
Total patients	861 (8%)	569 (5%)	5,282 (47%)	1,247 (11%)	3,204 (29%)	11,163 (100%)
Females	750 (87%)	489 (86%)	4,027 (76%)	985 (79%)	2,179 (68%)	8,484 (76%)
Males	111 (13%)	80 (14%)	1,255 (24%)	262 (21%)	1,025 (32%)	2,679 (24%)
Age at inclusion (years)	62 (48–75)	50 (37–61)	55 (40–69)	65 (52–75)	55 (39–72)	57 (42–71)
Age groups						
18–29	53 (6%)	73 (13%)	583 (11%)	55 (4%)	359 (11%)	1,123 (10%)
30–49	189 (22%)	204 (36%)	1,515 (29%)	216 (17%)	867 (27%)	2,991 (27%)
50–69	308 (36%)	220 (39%)	1,936 (37%)	481 (39%)	1,047 (33%)	3,992 (36%)
70+	311 (36%)	72 (13%)	1,248 (24%)	495 (40%)	931 (29%)	3,057 (27%)
Time to group definition (months)	23 (8–49)	14 (3–41)	12 (5–28)	-	-	-
Total FU time (months)	76 (47–104)	72 (41–99)	65 (37–95)	55 (28–87)	29 (12–60)	55 (26–88)
TSH at inclusion (mU/L)	0.08 (0.02–0.21)	0.06 (0.02–0.16)	0.26 (0.13–0.35)	0.18 (0.06–0.30)	0.27 (0.13–0.37)	0.24 (0.09–0.34)
<0.1	453 (53%)	359 (63%)	1,049 (20%)	395 (32%)	616 (19%)	2,872 (26%)
≥0.1	408 (47%)	210 (37%)	4,233 (80%)	852 (68%)	2,588 (81%)	8,291 (74%)
THR use during FU	384 (45%)	274 (48%)	263 (5%)	61 (5%)	78 (2%)	1,060 (9%)
ATD use during FU	384 (45%)	71 (12%)	182 (3%)	72 (6%)	59 (2%)	768 (7%)

FU, follow–up; ATD, antithyroid drugs; (S)HOT, (subclinical) hypothyroidism; SHT, subclinical hyperthyroidism; THR, thyroid hormone replacement; HT, hyperthyroidism.

**Figure 3:**
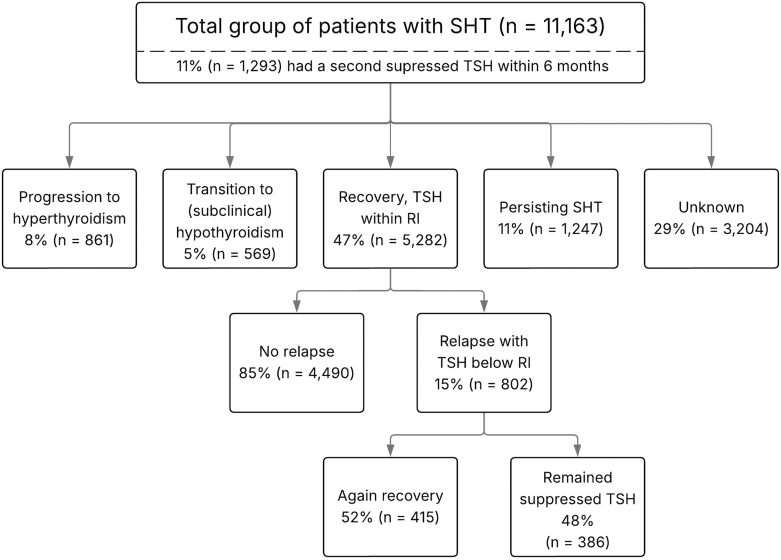
Flowchart of the different outcome pathways.

In the patients progressing to hyperthyroidism or transitioning to (subclinical) hypothyroidism, TSH levels at the start were below 0.1 mU/L for 53 and 63%, respectively, compared to only 20 and 32% of those in the recovery and persisting SHT groups. Use of thyroid hormone replacement and antithyroid drugs varied significantly across groups. In the Netherlands, block-and-replace regimens are frequently used, which could explain thyroid hormone replacement use. Forty-five percent of the patients who progressed to hyperthyroidism used thyroid hormone replacement during follow-up, while this was much less common (5%) in those who recovered. Similarly, antithyroid drug use was more prevalent in the progression group, with 45% of patients receiving these medications, compared to only 3% among those who recovered. This could partially be explained by relapses in the recovery group. Additional analysis showed that 15% (802 patients) of this group experienced a relapse, where their TSH levels fell back below the lower RI. This is summarized in Fig. 3. Although none of these patients developed hyperthyroidism (elevated FT4 concentrations), 8% (415) eventually normalized their TSH levels, while 7% (386) continued to have TSH levels below the RI.

A second low TSH measurement within 6 months was present in 11% (1,239) of the cases. Among these, 22% progressed to hyperthyroidism, 5% transitioned to (subclinical) hypothyroidism, 34% achieved recovery, and 37% had persisting SHT.

In a sensitivity analysis using a stricter TSH threshold of <0.1 mIU/L (grade 2 SHT), more individuals progressed to hyperthyroidism or hypothyroidism, and fewer recovered. Mean age was slightly lower, while sex distribution and follow-up duration were similar. Thyroid medication use was higher (Supplementary Table S3). To assess the influence of transient conditions such as thyroiditis, we performed a sensitivity analysis excluding patients with elevated CRP or ESR within 2 weeks of the initial low TSH measurement (Supplementary Table S4). The results were highly similar to those of the main analysis.

### Factors associated with recovery and progression

The logistic regression analysis identified factors associated with recovery and progression (Table 2). These analyses included only patients with known outcomes, using those with persisting SHT as the reference group. Younger age, male sex, and TSH levels at inclusion ≥0.1 mU/L significantly increased odds for recovery. Odds for progression to hyperthyroidism were higher in lower age groups, female sex, TSH levels at inclusion <0.1 mU/L, and when multiple comorbidities were present.

**Table 2:** Logistic regression analysis of factors associated with recovery of subclinical hyperthyroidism and progression to hyperthyroidism.

Variable	OR (95% CI)	Number per group (%)	
**Recovery vs persisting SHT**		**Recovery**	**Persisting**
Age group			
18–29	4.81 (3.51–6.68)	583 (11%)	55 (4%)
30–49	3.18 (2.59–3.91)	1,515 (29%)	216 (17%)
50–69	1.71 (1.47–2.00)	1,936 (37%)	481 (39%)
70+	Reference	1,248 (24%)	495 (40%)
Women	0.82 (0.70–0.96)	4,027 (76%)	985 (79%)
Men	Reference	1,255 (24%)	262 (21%)
TSH levels at inclusion, mU/L			
<0.1	0.50 (0.43–0.58)	1,049 (20%)	395 (32%)
≥0.1	Reference	4,233 (80%)	852 (68%)
Comorbidity score			
0 points	Reference	2,384 (45%)	431 (35%)
1–2 points	0.99 (0.84–1.15)	2,161 (41%)	598 (48%)
3–4 points	1.20 (0.96–1.48)	737 (14%)	218 (17%)
**Progression vs persisting SHT**		**Progression**	**Persisting**
Age groups			
18–29	1.81 (1.15–2.84)	53 (6%)	55 (4%)
30–49	1.65 (1.23–2.21)	189 (22%)	216 (17%)
50–69	1.10 (0.89–1.37)	308 (36%)	481 (39%)
70+	Reference	311 (36%)	495 (40%)
Females	1.69 (1.32–2.17)	750 (87%)	985 (79%)
Males	Reference	111 (13%)	262 (21%)
TSH levels at inclusion, mU/L			
<0.1	2.36 (1.97–2.83)	453 (53%)	395 (32%)
≥0.1	Reference	408 (47%)	852 (68%)
Comorbidity score			
0 points	Reference	307 (36%)	431 (35%)
1–2 points	1.20 (0.95–1.52)	382 (44%)	598 (48%)
3–4 points	1.62 (1.20–2.19)	172 (20%)	218 (17%)

CI, confidence interval; SHT, subclinical hyperthyroidism; OR, odds ratio.

The analysis in Table 2 was repeated with a TSH threshold of 0.05 mU/L instead of 0.1 mU/L in order to see whether complete TSH suppression would change the odds ratios. For recovery versus persisting, this resulted in an odds ratio of 0.58 (95% CI: 0.49–0.68), and for progression versus persisting, an odds ratio of 2.43 (95% CI: 1.99–2.97).

### Guideline adherence

Within the initial 6-month period, 32% of patients (3,572) underwent a follow-up TSH measurement. This percentage decreased to 26% (2,867) when the initial 6-week period was excluded and to 28% (3,142) when the initial 4-week period was excluded. As shown in Table 1, 3,204 (29%) of patients had no follow-up measurement. Regarding the measurement of inflammatory markers within the initial 6-month period, for 83% of patients (8,940) an ESR measurement was available, for 71% (7,628) a CRP measurement, and for 87% (9,407) a leukocyte count. However, in only 4% (428) of the cases was TRAb testing performed.

## Discussion

This study aimed to assess the incidence, natural course, and guideline adherence of SHT in primary care. This study showed an annual incidence of SHT of approximately 200 cases per 100,000 person-years, and in only a small subset of SHT patients, a progression to overt hyperthyroidism. Significant contributing factors of progression included lower TSH at baseline, female sex, and younger age. Despite recommendations for follow-up TSH measurements and TRAb testing, these were not present in the majority of patients.

In the literature, the prevalence of SHT is frequently reported, but incidence rates are rarely provided (16, 17). We found a stable annual incidence of ∼200 cases per 100,000 person-years between 2010 and 2015. A temporary increase was observed in 2016 and 2017, followed by a gradual increase in subsequent years. This rise coincides with the 2016–2017 Thyrax® shortage in the Netherlands, which may have indirectly influenced healthcare-seeking behavior or follow-up practices (18). The overlap in years is noteworthy but does not fully explain this incidence pattern, as we excluded individuals with thyroid medication use within the 2 years before inclusion. In addition, this estimate reflects the detection of biochemically defined SHT based on low TSH values, which may include individuals with non-thyroidal causes of low TSH, or healthy individuals who present with TSH levels below the reference range by chance, rather than having true hyperthyroid disease. As TSH testing is done on indication in general practice, this incidence reflects the rate at which GPs encounter new cases among those tested, not the population-wide incidence. While this limits direct international comparisons, it offers a valuable real-world perspective on how often SHT presents in routine primary care. The incidence rate per 100,000 person-years was around 30–50 in a previous study reporting on the incidence of SHT between 1993 and 2008, based on TSH measured in primary care (9). This lower number can be explained by the applied inclusion criterion in that study of two TSH measurements at least several months apart. Current guidelines differ on whether it is required for SHT diagnosis but recommend several measurements before considering treatment options (8, 19).

Our findings suggest that adherence to the Dutch primary care guideline, which is similar to other European guidelines, is suboptimal. Despite recommendations for follow-up TSH measurements every 3 months for the first year, only about a third of patients received this within the first 6 months (15). The limited follow-up may be due to factors such as perceived low risk of complications, the asymptomatic nature of the condition, or the complexity of tracking subclinical disease in general practice. Indeed, the low rate of thyroid medication use and relatively high baseline TSH concentrations in the group without follow-up data suggest that this group does not consist of the most clinically relevant or persistent cases. Despite frequent ESR and leukocyte testing, TRAb adherence was low (4%), suggesting a disregard for testing without signs of thyroiditis, potentially delaying Graves’ diagnosis. The findings of this study reinforce the necessity for enhanced adherence to the existing guidelines, as timely TSH and antibody testing can lead to identifying patients at higher risk for progression.

Consistent with previous studies, our results indicated that approximately 25% of the cases exhibited a TSH level below 0.1 mU/L at baseline, with a notably higher risk in women, which is also observed elsewhere (9, 11, 20, 21, 22). Our median age (57 years) aligns with prior studies (9, 11, 20, 21, 22). In our cohort, 47% of patients recovered, 11% persisted, and 8% progressed to overt hyperthyroidism over a median follow-up of 5 years. Vadiveloo *et al.* observed recovery in 32%, persistent SHT in 68%, and progression in 0.7% in 693 patients with SHT after 5 years using routine care data (9). Their inclusion criterion of a second suppressed TSH measurement in the first 6 months and taking the mode of multiple FT4 measurements in a year could have led to an underestimation of the amount of hyperthyroidism, since 19% of all patients after 5 years were on thyroid treatment, meaning that their FT4 might be within the RI because of the treatment. Consistent with our findings, older patients with TSH <0.01 mU/L also had lower odds of recovery.

Diez *et al.* reported a higher progression rate (45%) to overt hyperthyroidism, particularly among those with TSH <0.1 mU/L, and showed a 20% recovery rate in a small cohort of 75 patients followed for 5 years (20). Similarly, Das *et al.*, with a cohort of 323 patients and a follow-up of 2.5 years, found that 12% progressed to hyperthyroidism, with those with TSH <0.1 mU/L having a 3.4 times increased risk for progression (22). Our study aligns with these findings, reinforcing that very low TSH concentrations are strongly linked to progression. The etiology of SHT (e.g., multinodular goiter, Graves’ disease, or nodular disease) also predicted changes of progression or recovery in earlier studies but could not be assessed in our study (11, 23).

SHT is particularly relevant in women, who made up the majority of our study population. In terms of patient demographics, we found that women had higher odds of progression and lower odds of recovery compared to men, consistent with previous reports (16, 21). Rosario *et al.* followed 102 women, all >60 years and with a median follow-up of 3.5 years, and reported that 3–7% progressed, 24% recovered, and 70% persisted (24). Schouten *et al.* reported a progression rate of 5–8% per year in 96 patients with a median follow-up of 3.8 years (11). These findings, in combination with the overall low rates of diagnostic follow-up, such as TRAb testing, raise concerns about possible undertreatment or underrecognition. This is especially important for women because untreated SHT has been linked to an increased risk of atrial fibrillation and loss of bone mineral density. Our findings highlight the need for greater awareness of guideline adherence in women, and we aim to further investigate cardiovascular and skeletal outcomes in future studies within this cohort (https://doi.org/10.1186/ISRCTN15496928).

Based on our findings, some considerations for the follow-up of patients with subclinical hyperthyroidism (SHT) can be proposed, though further experimental research is needed to confirm these. Given the observed time range within which patients may progress to overt hyperthyroidism, a follow-up period of at least 5 years might be reasonable. For patients who demonstrated recovery, none showed progression to hyperthyroidism following sustained TSH normalization, suggesting that ongoing monitoring could potentially be discontinued after one or two normal TSH values. Older patients appeared more likely to experience persistent SHT, while younger individuals showed a tendency either to recover or progress, indicating that age alone may not be sufficient to guide follow-up. Women, regardless of age, and patients with a baseline TSH below 0.1 mU/L were more likely to progress and less likely to recover, indicating important factors in follow-up planning.

Our study has several limitations to be considered. First, the retrospective design and reliance on existing records may introduce biases due to incomplete documentation and variability in coding practices. For example, 28% of patients were lost to follow-up, and only 32% had a repeat TSH measurement within 6 months, reflecting inconsistent retesting intervals in routine care. In addition, we were unable to assess certain patient-level factors, such as symptom presentation or reasons for lack of follow-up. The PHARMO GP data only include data from general practice, meaning that some cases of SHT may have received thyroid medication that was not recorded, as it may have been prescribed by specialists rather than GPs. Our post hoc exploration in the PHARMO outpatient pharmacy data indicated that 2.6% of the baseline SHT group was on thyroid medication, suggesting that some cases could be attributed to exogenous rather than endogenous causes. Another limitation is the absence of T3 measurements, which are not recommended in Dutch primary care guidelines but would be required to exclude T3 thyrotoxicosis. In addition, a variety of TSH assays was used, but the effects on our results are expected to be minimal, as patients usually visit the same laboratory for routine follow-up laboratory measurements, and we previously found limited standardization differences in the low-concentrations range between commonly used TSH assays (25). In addition, medications such as glucocorticoids, opiates, metformin, and L-DOPA were not excluded, as these prescription data were not available in our dataset. Finally, although time-to-event analyses were considered, the irregular timing of TSH measurements in our real-world dataset would likely have introduced substantial measurement error; hence, we opted for fixed follow-up intervals as a more robust alternative. As testing was not protocolized, both the timing and frequency of follow-up varied, and recovery may have occurred earlier than documented.

Despite these limitations, our study has notable strengths. With the largest sample size to date for examining the natural course and incidence of SHT, our study offers robust statistical power for detecting differences across subgroups and provides a detailed view of guideline adherence in a real-world setting. By including only biochemically confirmed cases of SHT, we ensured an accurate representation of individuals with true SHT rather than relying on diagnostic coding alone. Furthermore, the Dutch PHARMO GP data have demonstrated generalizability to primary care settings across the Netherlands, enhancing the relevance of our findings to the context in which most SHT cases are managed (13).

In conclusion, this study provides valuable insights into the natural course and management of SHT within primary care. Our findings reveal a significant gap between guideline recommendations and real-world practice, suggesting that improvements in guideline adherence, particularly in follow-up and diagnostic testing, may optimize care and outcomes. The majority of patients in our study either recovered or maintained stable SHT, while a small subset progressed to overt hyperthyroidism, with lower TSH levels and female sex being significant predictors of progression. Our findings also highlight that sustained normalization of TSH may justify reduced follow-up in certain cases. Further studies are needed to confirm these recommendations and better define tailored follow-up protocols.

## Supplementary materials



## Declaration of interest

The authors declare no support from any organization for the submitted work; no financial relationships with any organizations that might have an interest in the submitted work in the previous 3 years; and no other relationships or activities that could appear to have influenced the submitted work.

## Funding

This work did not receive any specific grant from funding agencies in the public, commercial, or not-for-profit sectors for this research.

## Author contribution statement

SU was responsible for conceptualization, methodology, formal analysis, investigation, writing the original draft, writing review and editing, and visualization. WdE helped in conceptualization, methodology, investigation, and writing review and editing. JvdB was responsible for methodology and writing review and editing. AB helped in conceptualization and writing review and editing. PE was responsible for conceptualization, methodology, investigation, and writing review and editing. RN was responsible for methodology and writing review and editing. AH helped in conceptualization, methodology, supervision, and writing review and editing.

## References

[1] Cooper DS & Biondi B. Subclinical thyroid disease. Lancet 2012 379 1142–1154. (10.1016/S0140-6736(11)60276-6) 22273398

[2] Mitchell AL & Pearce SHS. How should we treat patients with low serum thyrotropin concentrations? Clin Endocrinol 2010 72 292–296. (10.1111/j.1365-2265.2009.03694.x) 19744106

[3] Biondi B & Cooper DS. Subclinical hyperthyroidism. N Engl J Med 2018 378 2411–2419. (10.1056/NEJMcp1709318) 29924956

[4] Collet TH, Gussekloo J, Bauer DC, et al. Subclinical hyperthyroidism and the risk of coronary heart disease and mortality. Arch Intern Med 2012 172 799–809. (10.1001/archinternmed.2012.402) 22529182 PMC3872478

[5] Gencer B, Collet TH, Virgini V, et al. Subclinical thyroid dysfunction and the risk of heart failure events an individual participant data analysis from 6 prospective cohorts. Circulation 2012 126 1040–1049. (10.1161/CIRCULATIONAHA.112.096024) 22821943 PMC3884576

[6] Cappola AR, Fried LP, Arnold AM, et al. Thyroid status, cardiovascular risk, and mortality in older adults. JAMA 2006 295 1033. (10.1001/jama.295.9.1033) 16507804 PMC1387822

[7] Donangelo I & Suh SY. Subclinical hyperthyroidism: when to consider treatment. Am Fam Physician 2017 95 710–716. 28671443

[8] Biondi B, Bartalena L, Cooper DS, et al. The 2015 European Thyroid Association Guidelines on diagnosis and treatment of endogenous subclinical hyperthyroidism. Eur Thyroid J 2015 4 149–163. (10.1159/000438750) 26558232 PMC4637513

[9] Vadiveloo T, Donnan PT, Cochrane L, et al. The thyroid epidemiology, audit, and research study (TEARS): Morbidity in patients with endogenous subclinical hyperthyroidism. J Clin Endocrinol Metab 2011 96 1344–1351. (10.1210/jc.2010-2693) 21346066

[10] Meyerovitch J, Rotman-Pikielny P, Sherf M, et al. Serum thyrotropin measurements in the community: five-year follow-up in a large network of primary care physicians. Arch Intern Med 2007 167 1533–1538. (10.1001/archinte.167.14.1533) 17646608

[11] Schouten BJ, Brownlie BEW, Frampton CM, et al. Subclinical thyrotoxicosis in an outpatient population-predictors of outcome. Clin Endocrinol 2011 74 257–261. (10.1111/j.1365-2265.2010.03908.x) 21044113

[12] Ursem SR, Boelen A, Bruinstroop E, et al. A systematic review of subclinical hyperthyroidism guidelines: a remarkable range of recommendations. Eur Thyroid J 2024 13 e240036. (10.1530/ETJ-24-0036) 38758966 PMC11227059

[13] Overbeek JA, Swart KMA, Houben E, et al. Completeness and representativeness of the PHARMO general practitioner (GP) data: a comparison with national statistics. Clin Epidemiol 2023 15 1–11. (10.2147/CLEP.S389598) 36636730 PMC9830053

[14] Lorenzo-Seva U. SOLOMON: a method for splitting a sample into equivalent subsamples in factor analysis. Behav Res Methods 2022 54 2665–2677. (10.3758/s13428-021-01750-y) 34918226 PMC9729132

[15] Van Lieshout J, Felix-Schollaart B, Bolsius E, et al. NHG-standaard schildklieraandoeningen (M31), 2013. (https://richtlijnen.nhg.org/standaarden/schildklieraandoeningen#volledige-tekst-subklinische-hyperthyreodie). Accessed on 2 March 2023. 23985246

[16] Madariaga AG, Santos Palacios S, Guillén-Grima F, et al. The incidence and prevalence of thyroid dysfunction in Europe: a meta-analysis. J Clin Endocrinol Metab 2014 99 923–931. (10.1210/jc.2013-2409) 24423323

[17] Carlé A, Andersen SL, Boelaert K, et al. Management of endocrine disease: subclinical thyrotoxicosis: prevalence, causes and choice of therapy. Eur J Endocrinol 2017 176 R325–R337. (10.1530/EJE-16-0276) 28274949

[18] Flinterman LE, Kuiper JG, Korevaar JC, et al. Impact of a forced dose-equivalent levothyroxine brand switch on plasma thyrotropin: a cohort study. Thyroid 2020 30 821–828. (10.1089/thy.2019.0414) 32188356

[19] Ross DS, Burch HB, Cooper DS, et al. 2016 American Thyroid Association Guidelines for diagnosis and management of hyperthyroidism and other causes of thyrotoxicosis. Am Thyroid Assoc 2016 26 1343–1421. (10.1089/thy.2016.0229) 27521067

[20] Díez JJ & Iglesias P. An analysis of the natural course of subclinical hyperthyroidism. Am J Med Sci 2009 337 225–232. (10.1097/MAJ.0b013e318187e16d) 19402203

[21] Strikić Đula I, Pleić N, Babić Leko M, et al. Epidemiology of hypothyroidism, hyperthyroidism and positive thyroid antibodies in the Croatian population. Biology 2022 11 394. (10.3390/biology11030394) 35336768 PMC8945477

[22] Das G, Ojewuyi TA, Baglioni P, et al. Serum thyrotrophin at baseline predicts the natural course of subclinical hyperthyroidism. Clin Endocrinol 2012 77 146–151. (10.1111/j.1365-2265.2012.04345.x) 22283624

[23] Rosario PW. The natural history of subclinical hyperthyroidism in patients below the age of 65 years. Clin Endocrinol 2008 68 491–492. (10.1111/j.1365-2265.2007.03030.x) 17803710

[24] Rosario PW. Natural history of subclinical hyperthyroidism in elderly patients with TSH between 0·1 and 0·4 mIU/L: a prospective study. Clin Endocrinol 2010 72 685–688. (10.1111/j.1365-2265.2009.03696.x) 20447066

[25] Ursem SR, Boelen A, Hillebrand JJ, et al. How low can we (reliably) go? A method comparison of TSH assays with a focus on low concentrations. Eur Thyroid J 2023 12 e230123. (10.1530/ETJ-23-0123) 37552779 PMC10503215

