# Supplementary material for: Natural course of subclinical hyperthyroidism in primary care in the Netherlands
Source: Eur Thyroid J. 2025 Nov 18;14(6):e250142. doi: 10.1530/ETJ-25-0142 (PMC12630534; doi:10.1530/ETJ-25-0142)
Supplement: Supplementary file 1 [file supplementary_materials.pdf]

Supplementary detailed method description

Incidence

For the incidence of SHT, all individuals of 18 years and older in PHARMO's GP data who were registered as being present in the GP practice (active) on January 1st of each calendar year and who were registered at least one year in the patient file were included. All individuals with SHT before January 1st of each calendar year were excluded. Additionally, patients using lithium (anywhere before SHT diagnosis, amiodarone or thyroid medication (2 years before SHT) were excluded. As we studied the incidence of biochemically defined SHT based on TSH values alone, this may include individuals with non-thyroidal causes of low TSH or healthy individuals falling below the reference range by chance, rather than reflecting true hyperthyroid disease. The observed incidence of SHT was determined among all individuals in the patient file with at least one year of data available, defined as the population at risk. At least one year of data availability was required to ensure each person had an equal chance of being marked as an incident person with SHT. The observed incidence was calculated by dividing the sum of individuals with incident SHT by the total number of person-time at risk in the patient file for both women and men during the calendar year. Person-time at risk in the patient file was defined as the time between January 1st of the year, or start follow-up in the GP practice, whatever came last, and diagnosis date of SHT, December 31st of the year or end of follow-up in the GP practice, whichever came first.

To evaluate whether the incidence of SHT in specific years deviated from the overall trend, we fitted negative binomial regression models using yearly counts of incident cases from 2010 to 2021. The log of person-years was included as an offset. A continuous variable for calendar year was included to model the overall linear time trend. Indicator variables for individual years were added to estimate deviations from this trend. Exponentiated coefficients were reported as incidence rate ratios (IRRs) comparing observed incidence in each year to the expected incidence based on the trend.

Study population for dataset

The study population consisted of patients identified through TSH measurements requested in primary care, excluding those with known thyroid diseases.

SHT was defined by a TSH concentration below the lower limit of the reference interval and an FT4 concentration within the reference interval at the same measurement or patients with a recorded ICPC code A91.07 (subclinical hyperthyroidism). Method specific reference intervals, as reported by the respective laboratories, were used for each TSH and FT4 measurement.

Exclusion criteria for SHT were: in the 2 years prior to inclusion (1), use of thyroid medication (ATC starting with H03), amiodarone (ATC C01BD01) or ever recorded use of lithium (ATC N05AN01) or (2) mention of ICPC codes: T85 (hyperthyroidism), T86 (hypothyroidism), A91.06 (subclinical hypothyroidism), A91.07 (subclinical hyperthyroidism) or T71 (thyroid malignancy).

Patients without available data for the study period or who could not be matched to controls were excluded from the analysis. In addition, subjects younger than 18 years of age at time of inclusion were excluded. In order to minimize the chance of thyroid disorders being related to pregnancy we excluded patients with SHT which were included three months before to one year after a registered pregnancy (ICPC codes W78 "Desired pregnancy" or W79 "Unwanted pregnancy").

If a pregnancy was registered in the follow-up TSH and FT4 values were also excluded in the lab dataset three months before to one year after a registered pregnancy. We performed a sensitivity analysis, requiring a second suppressed TSH measurement four weeks to six months after the initial TSH measurement for inclusion as subclinical hyperthyroidism.

Outcome measures

In this study, we identified the following subgroups:

1. Progression to overt hyperthyroidism, defined as FT4 levels above the upper limit of the reference interval during the follow-up period.
2. Progression to (subclinical) hypothyroidism, defined as a TSH level above the upper limit of the reference interval at any time during follow-up, but not in group 1.
3. Recovery, characterized by TSH levels returning to within the reference interval at any time during the follow-up, but not in group 1 or 2.
4. Persisting subclinical hyperthyroidism, defined as persistently suppressed TSH levels with FT4 levels within the reference interval throughout the follow-up period, but not in group 1-3.
5. Unknown, which group includes patients who were not included in the other groups due to missing TSH or FT4 measurements during follow-up.

For the above groups the TSH and FT4 concentrations in the first four weeks were excluded, since they were deemed too close to the inclusion date, as the half-life time of thyroxine is around 7 days and the Dutch primary care guideline recommends testing only after three months. We assessed the time to group definition, age, sex and TSH concentrations at inclusion. Additionally, we examined which patients started using thyroid hormone replacement (ATC codes starting with H03A) or antithyroid drugs (ATC codes starting with H03B). These comparisons were made across the entire available follow-up period, as well as with cut-off points at 2 years and 5 years.

We performed a sensitivity analysis for the recovery group in order to explore the number of patients that relapsed after being marked as recovered and excluding those from the analysis. In addition, we performed a sensitivity analysis using a stricter TSH threshold of <0.1 mIU/L (grade 2 SHT).

Statistical analysis

To compare subgroups, we utilized ANOVA for continuous variables and Chi-square tests for categorical variables. To identify factors associated with progression or recovery, we performed logistic regression analyses. The dependent variables in these models were recovery versus persisting subclinical hyperthyroidism and progression versus persisting subclinical hyperthyroidism. The independent variables included age, sex, TSH level at inclusion and comorbidity score. The comorbidity score was defined as a composite variable representing the sum of individual risk factors associated with cardiometabolic disease. Each identified risk factor contributed one point to the total comorbidity score. The risk factors were extracted for each case for one year before cohort entry date. They are as follows (1 point per comorbidity):

1. Hypertension: Defined by the presence of ICPC codes H86 or H87 or the use of antihypertensive medications (specifically antihypertensives [ATC C02], diuretics [ATC C03], beta-blocking agents [ATC C07], calcium channel blockers [ATC C08] or agents acting on the renin-angiotensin system [ATC C09]).

2. Hypercholesterolemia: Defined by either LDL cholesterol level >2.6 mmol/L or use of statins (ATC C10).
3. Kidney Disease: Defined by either: estimated glomerular filtration rate (eGFR) <60 mL/min/1.73 m^2 or albumin-to-creatinine ratio (ACR) >3 mg/mmol.
4. Diabetes Mellitus: Defined by the presence of ICPC code T90 or the use of diabetes medications (ATC A10).

All statistical analyses were conducted using R version 4.2.2 (2022-10-31 ucrt). The following R packages were used for the analysis: car, dplyr, ggplot2, lubridate, multcomp, readr, and tidyr.

Supplementary Table S2. Incidence rate ratios for the incidence of subclinical hyperthyroidism in each calendar year compared to the estimated trend.

Effect	IRR	95% CI
Per-year linear trend	1.05	1.03–1.06
2011	0.97	0.77–1.23
2012	1.00	0.80–1.25
2013	0.97	0.79–1.19
2014	0.90	0.74–1.10
2015	0.87	0.76–1.00
2016	1.24	1.08–1.43
2017	1.21	1.02–1.44
2018	0.93	0.79–1.11
2019	0.89	0.71–1.13
2020	0.91	0.71–1.16
2021	0.92	0.71–1.20

IRR, incidence rate ratio; CI, confidence interval. IRRs were estimated using negative binomial regression models with an offset for log(person-years). The model included a linear time trend variable, and each year was tested as an indicator for deviation from this trend. IRRs >1 indicate higher incidence than expected based on the trend, and IRRs <1 indicate lower incidence.

Supplementary Table S3. Characteristics of subgroups of patients with subclinical hyperthyroidism with TSH <0.1 mU/L at inclusion

**Group→**<br>Variable↓	**Progression to hyperthyroidism**	**Transition to (subclinical) hypothyroidism**	**Recovery**	**Persisting SHT**	**Unknown**	**Total**
Percentage of total (%, N)	16% (453)	13% (359)	37% (1,049)	14% (395)	21% (616)	100% (2,872)
Female (%, N)	88% (399)	85% (305)	83% (871)	83% (328)	68% (419)	76% (2183)
Male (%, N)	12% (54)	15% (54)	17% (178)	17% (67)	32% (197)	24% (689)
Age at inclusion (years, IQR)	59 (45-73)	50 (39-63)	50 (37-63)	66 (53-77)	54 (41-70)	54 (42-49)
Time to group definition (months, IQR)	20 (6-46)	15 (2-41)	14 (4-33)	8 (3-19)	-	-
Total FU time (months, IQR)	78 (48-106)	72 (51-99)	68 (41-96)	59 (28-88)	33 (15-61)	61 (32-93)
TSH at inclusion (mU/L, IQR)	0.02 (0.01-0.05)	0.02 (0.01-0.06)	0.03 (0.01-0.06)	0.03 (0.01-0.06)	0.03 (0.01-0.06)	0.03 (0.01-0.06)
Thyroid hormone replacement use during FU (%, N)	48% (219)	47% (167)	11% (115)	7% (29)	7% (45)	20% (575)
ATD use during FU (%, N)	57% (257)	15% (54)	11% (113)	10% (39)	7% (44)	18% (507)

FU, follow-up, ATD, antithyroid drugs, SHT, subclinical hyperthyroidism

Supplementary Table S4. Characteristics of subgroups of patients with subclinical hyperthyroidism without increased ESR of CRP in the first two weeks after inclusion

**Group→**<br>Variable↓	**Progression to hyperthyroidism**	**Transition to (subclinical) hypothyroidism**	**Recovery**	**Persisting SHT**	**Unknown**	**Total**
Percentage of total (%, N)	8% (748)	5% (460)	47% (4,258)	12% (1,072)	28% (2,544)	100% (9,082)
Female (%, N)	88% (658)	87% (400)	78% (3,321)	80% (858)	71% (1,806)	77% (6,993)
Male (%, N)	12% (90)	13% (60)	22% (937)	20% (214)	29% (738)	23% (2,089)
Age at inclusion (years, IQR)	62 (48-74)	50 (36-60)	54 (40-54)	65 (52-75)	53 (36-69)	56 (41-70)
Time to group definition (months, IQR)	23 (8-49)	16 (4-44)	12 (5-28)	-	-	-
Total FU time (months, IQR)	78 (47-105)	72 (44-100)	66 (37-96)	56 (28-87)	31 (13-60)	57 (27-89)
TSH at inclusion (mU/L, IQR)	0.07 (0.02-0.21)	0.06 (0.02-0.16)	0.26 (0.13-0.35)	0.18 (0.06-0.30)	0.28 (0.13-0.37)	0.23 (0.08-0.34)
TSH <0.1 mU/L at inclusion (%, N)	54% (402)	64% (296)	21% (879)	32% (345)	20% (510)	27% (2,428)
TSH ≥0.1 mU/L at inclusion (%, N)	46% (346)	36% (168)	79% (3,379)	68% (727)	80% (2,034)	73% (6,654)
Thyroid hormone replacement use during FU (%, N)	47% (350)	47% (218)	5% (227)	5% (54)	2% (57)	10% (906)
ATD use during FU (%, N)	45% (338)	13% (61)	4% (152)	6% (62)	2% (45)	7% (658)

FU, follow-up, ATD, antithyroid drugs, SHT, subclinical hyperthyroidism
